# Tumour assessment of ROR1 levels in various adult leukaemia and lymphoma types

**DOI:** 10.1371/journal.pone.0313026

**Published:** 2024-11-04

**Authors:** Manuel A. Silva, Shuntae Williams, Sylvie Hauert, Benjamin Ovadia, Indu Gupta, Lorenz Waldmeier, Yarúa Jaimes, Hytham Al-Masri

**Affiliations:** 1 Department of Translational Medicine and Clinical Pharmacology, Boehringer-Ingelheim GmbH, Ingelheim am Rhein, Hesse, Germany; 2 Hematogenix, Chicago, Illinois, United States of America; 3 NeoGenomics, Fort Myers, Florida, United States of America; 4 NBE Therapeutics AG, Basel, Switzerland; Duke University Medical Center: Duke University Hospital, UNITED STATES OF AMERICA

## Abstract

Receptor tyrosine kinase-like orphan receptor 1 (ROR1) is a tumour target currently used for the development of novel therapeutic modalities, such as antibody-drug conjugates, chimeric antigen receptor T-cell therapies, and others. Success of these new drugs depends on the selection of relevant indications based on ROR1 tumour prevalence, staining heterogeneity, and subcellular localization, among other parameters. We investigated ROR1 immunophenotype using validated antibody clones for immunohistochemistry (IHC) and flow cytometry (FC), analyzing 292 tumour specimens from 7 haematological malignancies and triple negative breast cancer (TNBC) as a reference solid tumour indication. ROR1 prevalence varied significantly across distinct tumour types, showing 100% of ROR1 positivity in all chronic lymphocytic leukaemia (n = 48) and hairy cell leukaemia (n = 14) specimens analyzed via FC with ranges between 1.1–99.8% and 0.8–62.1%, respectively. Samples analysed via IHC showed ROR1 membrane/cytoplasmic positivity in 44% of mantle cell lymphoma tumour samples (n = 27; H-score range: 10–285 in positive cases); 30% in TNBC (n = 46; H-score range: 1–200); 15% in diffuse large B-cell lymphoma (n = 45; H-score: 40–250); and 11% in follicular lymphoma (n = 34; H-score: 2–300). Finally, all acute myeloid leukaemia (n = 52) and most T-cell non-Hodgkin lymphoma (n = 31/32) tested samples were negative for ROR1 via IHC. In conclusion, ROR1 shows a heterogeneous tumour cell expression profile across multiple leukaemias and lymphomas, making it a tumour target that would require different patient selection strategies to develop novel therapeutic modalities.

## Introduction

ROR1 is a transmembrane member of the tyrosine kinase-like orphan receptor (ROR) family that functions as a primary receptor for Wnt5a, and is widely expressed during embryonic development [1, 2]. ROR1 is expressed in the form of four transcript variants, three of which code for proteins: variant 1 (v1) encoding a full transmembrane protein consisting of 937 amino acids (aa); variant 2 (v2) encoding for a protein that carries a signal peptide but missing the transmembrane and intracellular domain; and variant 3 (v3) encoding for a protein missing the signal peptide resulting in intracellular localization and being the most highly expressed isoform in both normal and tumoural adult investigated tissues [3].

In the context of cancer, ROR1 protein levels have been extensively reported using flow cytometry (FC) in multiple haematological tumour types, including acute lymphoblastic leukaemia (ALL), chronic lymphocytic leukaemia (CLL), diffuse large B-cell lymphoma (DLBCL), follicular lymphoma (FL), mantle cell lymphoma (MCL), marginal zone lymphoma, and hairy cell leukaemia (HCL) [1, 4–6]. ROR1 prevalence via immunohistochemistry (IHC) in formalin-fixed paraffin-embedded (FFPE) tumour tissue samples have been widely described in multiple solid tumour indications using various antibodies (Ab) [7, 8]. Some of these Ab such as anti-ROR1 clone 4A5 displayed mainly cytoplasmic staining, which is not related to the expected cell-membrane ROR1 v1 expression pattern [7, 8]. Despite the low protein levels of ROR1 detected in some normal human tissues [1, 2, 7], ROR1 has been profiled as a promising tumour target for the development of new therapeutic modalities due to its role enhancing tumour cell growth and survival, potentiating epithermal growth factor receptor signaling and promoting epithelial-mesenchymal transition and metastasis [1, 2, 9]. Moreover, the prominent ROR1 membrane pattern observed in tumour cells, which should reflect v1 protein expression, has supported the development of an extensive variety of new immune-based treatments [1, 10]. These treatments include but are not limited to: antibody-drug conjugates (ADC) such as Zilovertamab Vedotin (NCT04504916 trial, Merck & Co) and NBE-002 (NCT04441099, NBE Therapeutics AG) in solid tumours, chimeric antigen receptor genetically modified T-cell (CAR-T) therapies in select ROR1 positive cases from haematological and solid tumour indications (NCT02706392, Fred Hutchinson cancer research Center), and a bi-specific CD3 T-cell engager (TCE) NVG-111 in ROR1 positive patients from haematological and solid tumour indications (NCT04763083, NovalGen Ltd).

Considering the limited amount of data published on haematological indications investigating morphologically intact tumours from lymph nodes or bone marrow [9], we performed this prevalence with a well characterized anti-ROR1 clone 6D4 for IHC [7], using commercially available FFPE tissue samples. We investigated acute myeloid leukaemia (AML) in biopsy and aspirate clot bone marrow tumours, as well as DLBCL, FL, MCL, and T-cell non-Hodgkin lymphoma (T-NHL) in respective FFPE tumoural lymph nodes, via IHC. Using flow cytometry (FC) with a AF647-labelled Hu4-2-17-LC clone, we analyzed whole blood and frozen samples from CLL and HCL cases to confirm previously published ROR1 prevalence [4–6], adding more information to the infrequent HCL type that accounts for 2% of all leukaemias [5, 11]. Finally, to bridge a comparison of ROR1 levels between haematological and solid tumours, we included a cohort of triple negative breast cancer (TNBC) FFPE tumour tissues as a reference solid tumour type profiled with a significant fraction of ROR1^+^ tumour cells [7].

## Methods

### FFPE specimen analysis via IHC

An anonymized cohort of 46 FFPE tissue blocks of invasive ductal TNBC, collected from surgical resections from female patients (age range: 40–92 years old), were provided by NBE Therapeutics to NeoGenomics. 4–5 μm serial sections were generated to be used within 30 days as recommended by tissue stability guidelines and stored at room temperature. One section was processed via H&E for a pathology quality check on morphology, for diagnosis and confirmation that each sample had more than 100 viable tumour cells. Additional serial slides were stained with the mouse anti-ROR1 clone 6D4 carrying a rabbit Fc component (NBE Therapeutics reagent) or a negative rabbit isotype control (Leica PA0777) at a concentration of 0.55 μg/mL, using Ventana ADB250 Ab diluting buffer solution. The following IHC protocol for the Leica Bond III autostainer was established: slides were baked for 60 minutes at 56–60°C and then dewaxed at 72°C using Leica AR9222 reagent. This was followed by antigen retrieval with Epitope Retrieval Solution 2 (ER2, pH 9.0; Leica # AR9640) for 20 minutes at 100°C and washed for 5 minutes with Leica AR9590 solution. A peroxidase block was performed afterwards for 5 minutes using the Leica DS9800 kit, followed by incubation with anti-ROR1 clone 6D4 or negative isotype control for 30 minutes, and then a polymer incubation using the DS9800 kit for 8 minutes. Slides were then washed with AR9590 solution for 4 minutes, incubated with DAB from the DS9800 kit for 10 minutes, and stained with Hematoxylin from the same kit for 5 minutes. This IHC protocol was optimized using the following 4 TNBC sample with known ROR1 levels: B2494-Tc15, AVD-26GZQ-9262A, AVD-26GZQ-8081A, and AVD-26GZQ-1272A. These 4 cases were assessed by a pathologist to confirm expected ROR1 staining pattern and lack of unspecific background. All ROR1 stained slides from our TNBC cohort were evaluated for a membranous and/or cytoplasmic staining pattern by a pathologist using a brightfield microscope. The percentage of viable ROR1+ tumour cells were recorded on the level of staining intensity: scoring 0 as nondetectable; +1 weak (translucid); +2 moderate (opaque); and +3 strong (solid) staining. H-scores (0–300) were then calculated in all ROR1+ cases using the following formulae: 3 x percentage of +3 staining intensity + 2 x percentage of +2 staining intensity + 1 x of percentage of +1 staining intensity. This is a standard method to present preclinical IHC data and extensively used -for example- to show PD-L1 levels in different tumour types [12, 13], relevant biomarker assessment in oncology.

Hematogenix optimized and validated the ROR1 IHC assay on the Leica BOND RX Automated Stainer to investigate anonymized haematological FFPE tumour samples provided by Hematogenix biobank from leftover tissues used for routine diagnostic testing and accessed for research. A well-characterized FFPE ovarian tumour sample, with +3 membranous staining pattern, was provided to Hematogenix as a positive control to validate the IHC protocol and assure proper assay performance. In brief, DAB (3,3’-Diaminobenzidine) chromogen immunohistochemistry staining was performed on the BOND RX Automated Stainer (Leica Biosystems) with BOND polymer refine detection kit (Leica # DS9800). FFPE tissue sections (4–5 μm) were antigen-unmasked with Epitope Retrieval Solution 2 (Leica # AR9640) for 30 minutes at 100°C. The slides were blocked for endogenous peroxidase activity with Peroxide Block for 5 minutes using Leica DS9800. Sections were incubated with anti-ROR1 clone 6D4 Rabbit-Fc (0.55 ug/ml) provided by BI or negative isotype control (Leica PA0777 Rabbit Isotype), for 60 minutes at room temperature, followed by HRP labeled polymer for 8 minutes, and visualized with hydrogen peroxide substrate and DAB chromogen for 10 minutes. Slides were counterstained with hematoxylin and manually coverslipped. Upon ROR1 IHC assay establishment, FFPE tumour samples with diagnosis confirmed by a Hematogenix pathologist were selected from the following haematological indications including 52 AML cases (15 having myelodysplastic-related changes and 3 monocytic differentiation); 45 DLBCL; 27 MCL; 34 FL; and 31 T-NHL (no subtype information available) as indicated in Table 1. Manual scoring by a pathologist was performed on ROR1 IHC, as described above, and percentage of viable ROR1+ tumour cells, as well as H-scores, were determined.

**Table 1:** Clinical data and tissue type from each haematological disease.

		Indication						
		AML	DLBCL	MCL	FL	T-NHL	CLL	HCL
**Total cases**		52	45	27	34	31	48	14
**Age range (years)**		32–89	27–87	54–88	42–92	22–80	48–93	49–88
**Sex**	**women**	23	23	6	19	6	17	2
	**men**	29	22	21	15	25	31	12
**Tumour tissue origin**		BM^a^	LN				PB/PBMC	
**Tissue format**		FFPE					Fresh^1^ & Frozen^2^	

BM: bone marrow (a: paired biopsy and aspirate clot); LN: surgically resected lymph nodes; PB: peripheral blood (1); PBMC: peripheral blood mononuclear cells (2)

### Peripheral blood specimen analysis via FC

As indicated in Table 1, fresh peripheral blood from patients diagnosed with CLL (n = 42) and frozen peripheral blood mononucleare cells (PBMC) from HCL cases (n = 14) or CLL cases (n = 6) were analysed at Hematogenix using FC. These anonymized samples were provided by Hematogenix biobank as left over from routine diagnostic testing or therapeutic procedures that otherwise would be destroyed, which were accessed for research purposes on 05/12/2022. As shown in S1A Fig, the panel is composed of two tubes, with identical antibodies except for the ROR1 which is present in tube 2 but not in the first tube. After comparing multiple fluorochrome-labelled and unlabelled anti-human ROR1 clones (S1B Fig), the mouse anti-ROR1 Hu4-2-17-LC antibody conjugated to AF647 was selected for the FC assay due to that our 10-plex flow cytometry assay setting will not accommodate a secondary detection step. ROR1 staining at any intensity (including bright or dim) was considered positive based on a negative isotype control assessment. In brief: frozen PBMC from each case were thawed, resuspended in PBS azide, and 100 μl of cell suspension was added to each tube of the panel shown in S1A Fig. The tubes were then centrifuged, vortexed, and incubated in the dark for 15 minutes to complete the staining process. Fresh peripheral blood samples were lysed using the TQ-Prep^™^ Workstation with the IMMUNOPREP Reagent System (Beckman Coulter), washed, and the white blood cells were resuspended in PBS azide. Afterwards, 100 μl of cell suspension was added to each tube containing the labeled Ab. The tubes were then centrifuged, vortexed, and incubated in the dark for 15 minutes. Once the staining step was completed, samples were washed and resuspended in PBS azide and fixed with a buffer from the IMMUNOPREP Reagent System. The samples were then acquired on the Navios^™^ EX Flow Cytometer (Beckman Coulter) and analyzed using the FCS Express De Novo software (version 6). Dead cells, cell aggregates, and debris were excluded from the analysis.

Afterwards, cells of interests were gated using CD45+ and CD19+. A diagnostic marker panel (S1A Fig) was used to identify tumoural B-cell subsets positive for ROR1. This diagnostic panel included CD19 and CD20 as pan B-cell markers, and CD45 as an additional B-cell marker. Kappa to Lambda ratios were evaluated for light chain analysis, with a ratio exceeding the normal range of 0.5–3.0 indicating the presence of a clonal process. Malignant B-cells are not only light chain restricted, but also deviate from normal B-cell marker expression, showing immunophenotypic aberrancies. In addition to positivity for lineage B-cell markers and clonality, CLL is defined as CD5+ and CD22+, while HCL is defined as CD5-, CD25+, and CD103+ [14, 15].

NeoGenomics and Hematogenix are Clinical Laboratory Improvement Amendments (CLIA) and College of American Pathologist (CAP) certified organizations assuring that the work was performed according to the highest standards compliant with US regulations covering quality and data privacy.

### Statistical analysis

For each indication, the percentage of ROR1 tumour positive cases was computed. To investigate if the ROR1 status (positive vs negative) was an independent parameter when comparing all indications, Fisher’s exact test was applied. The test was also applied to investigate pairwise differences. The *p*-values for these pairwise test comparisons were adjusted using Bonferroni correction.

## Results

### Heterogeneous ROR1 prevalence in tumour tissues by immunohistochemistry

**Fig 1:**
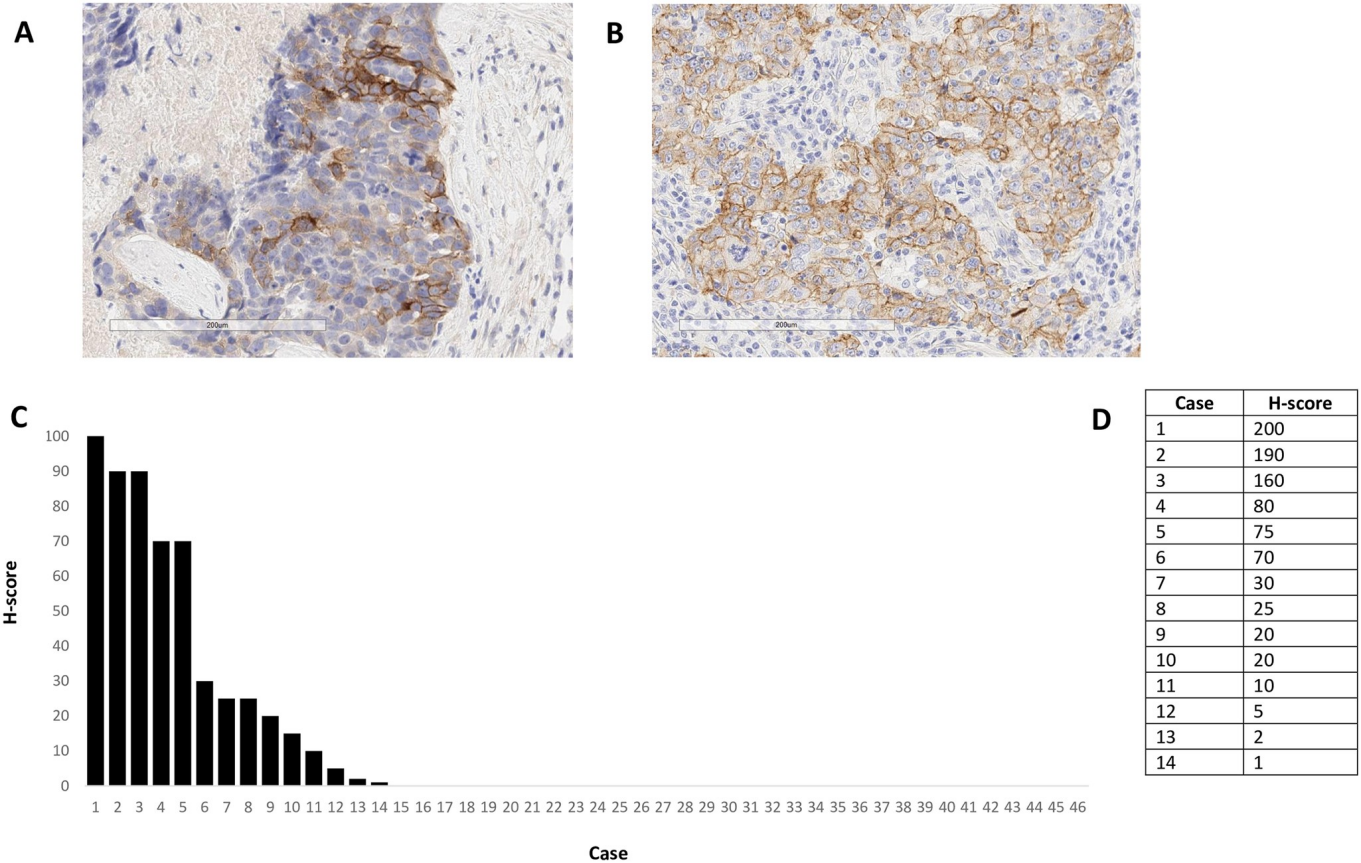
ROR1 IHC and scores in TNBC cases. A) ROR1 representative image from one of 4 TNBC cases used to optimize the IHC protocol in Leica BOND III instrument showing mainly tumour membrane staining pattern. B) ROR1 representative image from one of the 46 analyzed TNBC cases showing tumour cytoplasmic/membrane staining pattern. C) Waterfall showing % of ROR1+ tumour cells in all 46 cases. D) Table presenting ROR1 H-scores in all 14 ROR1+ cases.

**Fig 2:**
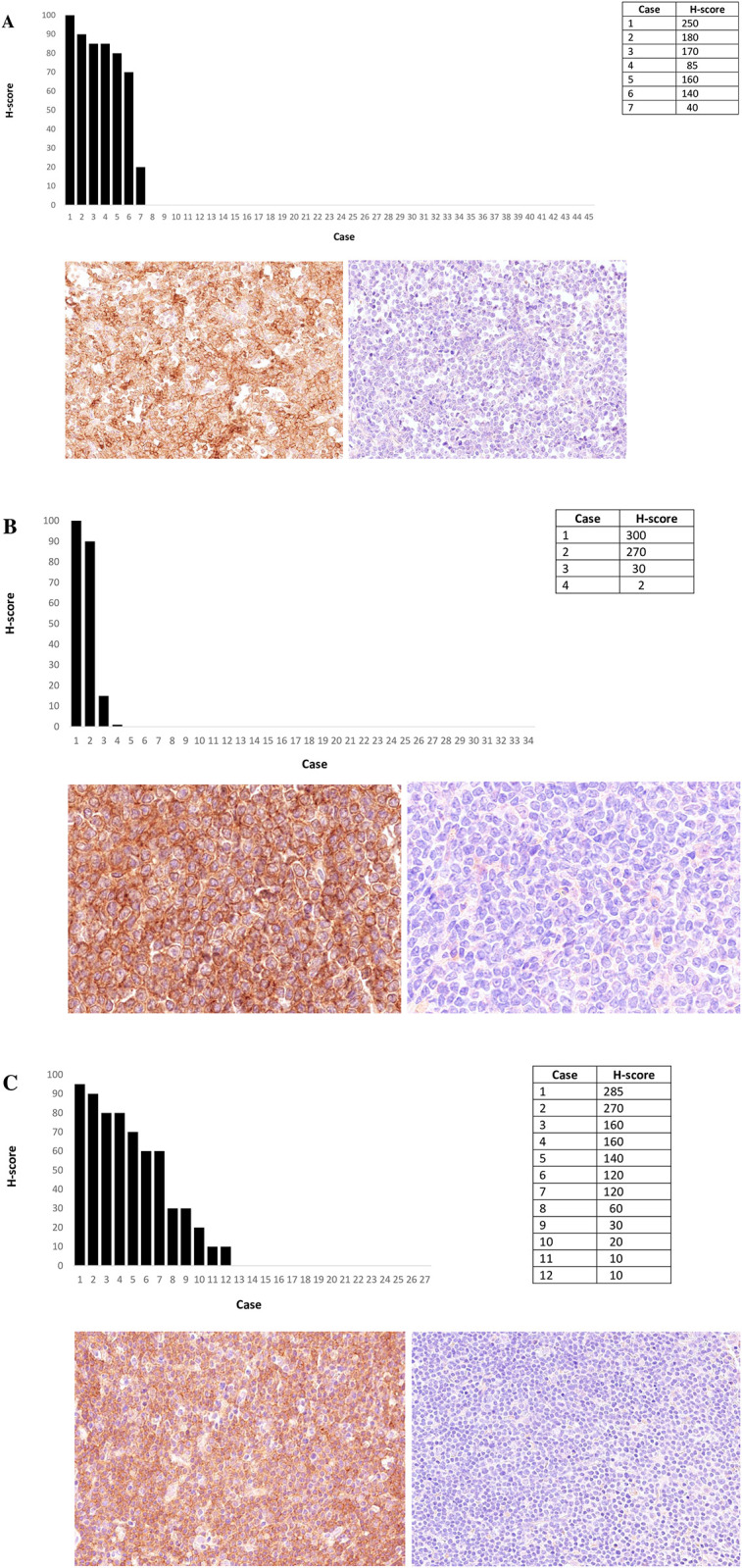
ROR1 IHC results in DLBCL, FL and MCL. A) Percentage of ROR1+ tumour cells in DLBCL (n = 45) including ROR1 IHC and isotype control 20X images from the case with the highest H-score of 250. B) percentage of ROR1+ tumour cells in FL (n = 34) including ROR1 IHC and isotype control 20X images from the case with the highest H-score of 300. C) Percentage of ROR1+ tumour cells in MCL (n = 27) including also ROR1 IHC and isotype control 20X images from the case with the highest H-score of 285.

ROR1 IHC staining pattern and prevalence results from TNBC are shown in Fig 1. ROR1 positive cases showed an expected tumoural cytoplasmic/membranous staining pattern. A staining pattern comparison between two different cases, Fig 1A from the assay validation group and Fig 1B from the analysed cohort, exemplifies the heterogeneity of ROR1 positivity found across various tumour samples. In our cohort, 30% (14/46) of the samples were ROR1 positive, including H-scores ranging from 1–200. The one case showing 100% of ROR1 positive tumour cells had the highest H-score of 200. When looking at our investigated haematological indications, ROR1 IHC results showed no staining in any of the 52 AML samples and 31 of the 32 T-NHL analysed samples. Only 1 T-NHL case showed ROR1 membrane tumour positivity with an H-score of 30. ROR1 IHC in each of the 52 AML samples was performed with a paired bone marrow core biopsy and aspirate clot tissue section on the same slide to investigate if bone marrow decalcification may have had an impact in staining. No ROR1 positive tumour cells were detected in either bone marrow core biopsies or aspirate clot tissue. ROR1 IHC results from other indications such as DLBCL, FL and MCL are shown in Fig 2. The ROR1 staining pattern in DLBCL, FL and MCL positive cases was cytoplasmic/membranous, as expected. Representative images from cases with highest H-scores per indication are shown in Fig 2. In terms of prevalence, ROR1 positivity in tumoural DLBCL lymph nodes was detected in 15% (7/45) of the cases, presenting H-scores ranging from 40 to 250 in these 7 positive samples (Fig 2A). The case with 100% of ROR1 positive tumour cells presented the highest H-score of 250. Results from tumoural FL samples showed ROR1 prevalence in only 11% (4/34) of the cases with H-scores ranging from 2 to 300 (Fig 2B). The case with 100% of ROR1 positive tumour cells showed the highest H-score of 300. Tumoural MCL lymph nodes presented a higher ROR1 prevalence of 44% (12/27) in our analysed cohort, including H-scores ranging from 10 to 285 (Fig 2C). The case with the highest ROR1 positive tumour cell score of 95% showed the highest H-score of 285.

### High prevalence of ROR1 via flow cytometry in CLL and HCL

**Fig 3:**
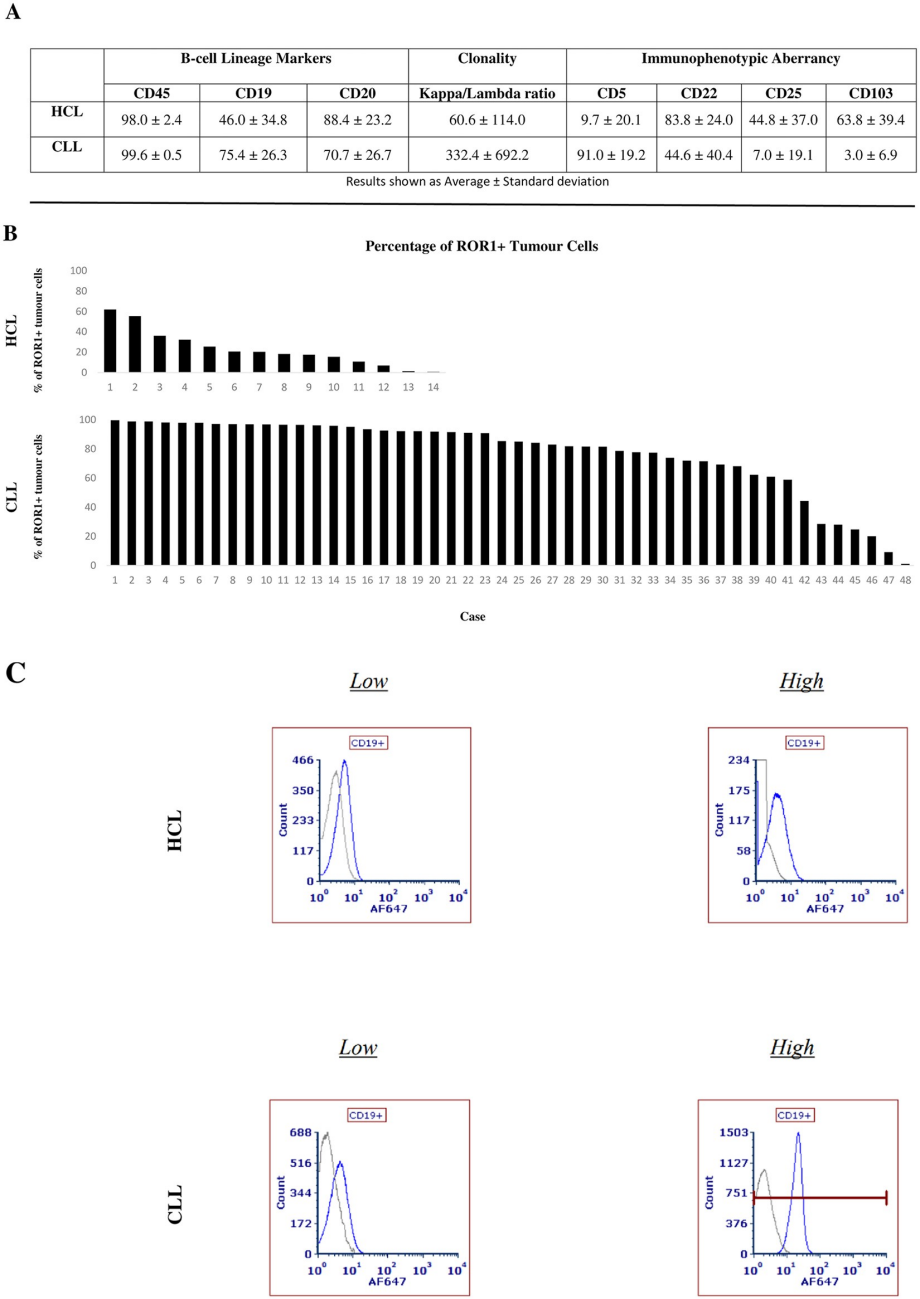
FC data summary in CLL (n = 48) and HCL (n = 14) cases. A) Showed percentage of positive cells for B-cell lineage, clonality and immunophenotypic aberrancy markers. B) Presents water fall graphs for ROR1 membrane tumour cell positivity in both indications. C) Histograms showing representative cases with high and low ROR1 levels in HCL and CLL, respectively.

Results showing percentage of cells according to B-cell lineage marker, clonality and immunophenotypic aberrancy in CLL and HCL cohorts are presented in Fig 3A. Tumour cells were initially gated by their CD45 immunophenotype, followed by CD19 positivity. The other pan-B cell marker, CD20, was expressed at 88.4% and 70.7% in HCL and CLL, respectively. When looking at clonality, both HCL and CLL showed high Kappa/Lambda ratio averages of 60.6 and 332.4, respectively, although with high heterogeneity. Regarding immunophenotypic aberrancies relevant for each disease, HCL showed a low 9.7% of CD5 positive cells, while expressing 44.8 and 63.8% of CD25 and CD103 positive cells, respectively. The CLL cohort presented 91.0 and 44.6% of CD5 and CD22 positive cells, respectively. ROR1 surface staining results are shown in Fig 3B. All CLL and HCL cases showed ROR1 positivity ranging from 1.1–99.8% and 0.8–62.1%, respectively. In addition, ROR1 values from frozen and fresh CLL samples were similar with 77.0 and 77.3% of positive tumour cells, respectively. Representative histograms for HCL and CLL with high and low ROR1 counts are presented in Fig 3C.

### Statistical comparison among indications

**Fig 4:**
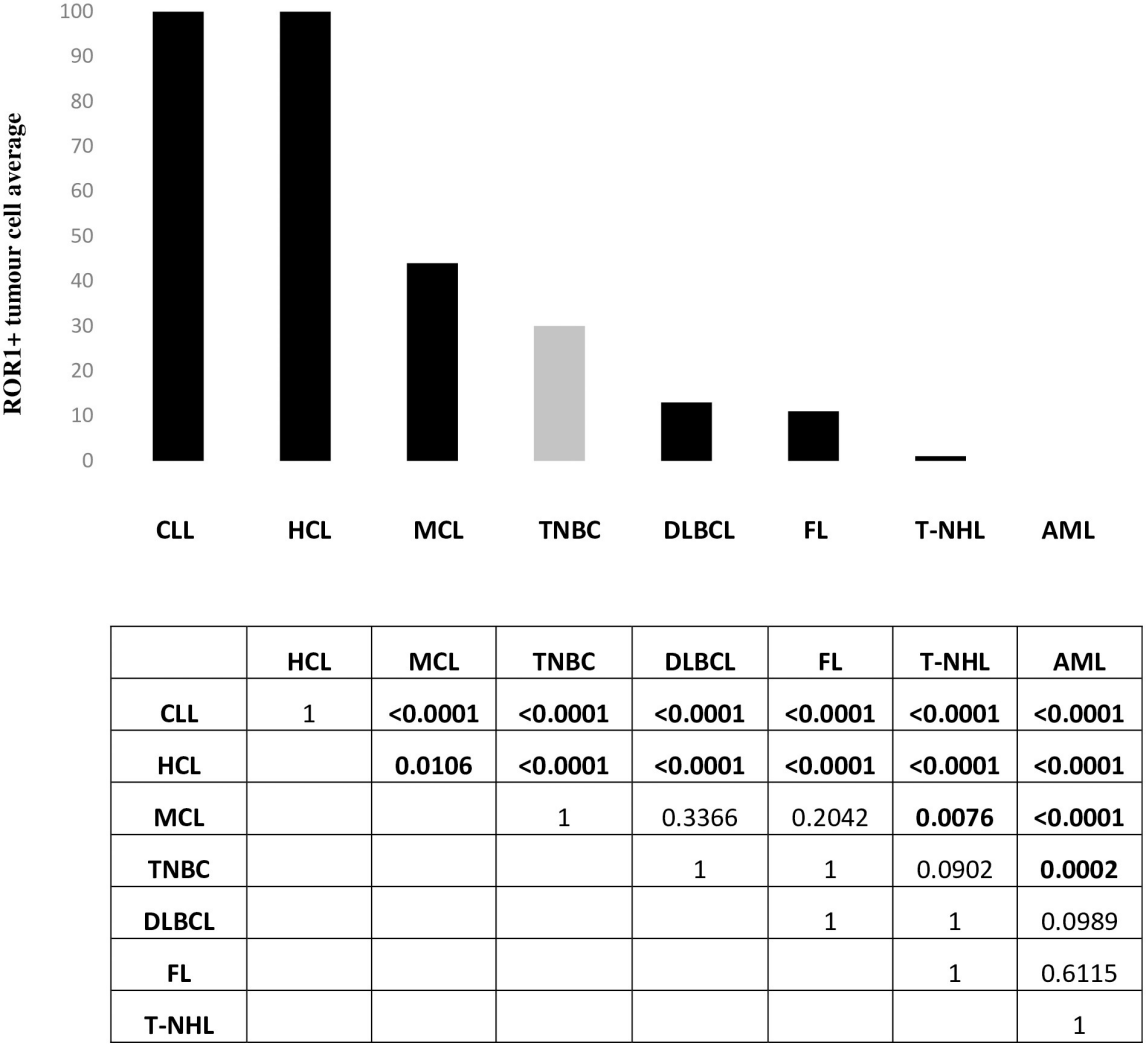
Comparison of ROR1 positive tumour cell averages in all analyzed haematological indications (black bars) and TNBC (gray bar: Reference solid tumour indication). Table showed Bonferroni corrected *p*-values for pairwise comparisons indicating significant differences in bold for *p* < 0.05.

ROR1 positive/negative tumour cell status was the common parameter available from both FC and IHC analyses to compare all investigated haematological cancer types, including our reference solid tumour indication TNBC. When statistical analysis was performed for this parameter, Fisher’s exact test determined a *p*-value of 0.0004998, indicating that the positive/negative ROR1 status was an independent parameter across all analysed indications. Averages of ROR1 positive cases per indications are shown in Fig 4. CLL and HCL cases showed significant differences with *p*-values < 0.05 when a pairwise comparison of their ROR1 positive/negative status was performed versus all other indications (Fig 4). In addition, MCL cases showed significant differences when pairwise comparisons were performed vs AML and T-NHL cases, respectively. Finally, TNBC cases showed significant differences versus AML cases.

## Discussion

Our study confirms a heterogeneous ROR1 prevalence profile across different haematological malignancies of both lymphoid and myeloid origins, with CLL and HCL showing the highest ROR1 positivity. There was significantly lower ROR1 prevalence in all other indications such as MCL, DLBCL, FL, as well as no ROR1 prevalence at all in AML and scant amounts in T-NHL. TNBC, our selected solid tumour indication for its relevant ROR1 prevalence, was in the middle of all 7 haematological tumour types analyzed as a reference point, being significantly lower in ROR1 positivity than CLL and HCL, but higher than in AML and T-NHL.

The expected ROR1 membranous/cytoplasmic staining pattern detected in all samples analyzed via IHC using clone 6D4 should reflect the presence of the expression of v1 and other variants in these tumour tissues as previously discussed by others [7]. Therefore, a more refined tumour membrane assessment, specific to v1 expression, will require a v1 specific anti-ROR1 Ab. This reagent will be key to identify v1-specific ROR1 positive cases in MCL, DLBCL and FL where ROR1 positivity showed average medium-to-low levels of 44, 15 and 11%, respectively. Moreover, our membranous/cytosolic ROR1 IHC data of 15% in DLBCL was much lower than the pure cytosolic staining in 83% of DLBCL cases described in a previous IHC study [9]. This difference between both studies might be explained by the usage of a polyclonal anti-ROR1 Ab in this previous publication that may have captured the protein isoform belonging to ROR1 expression mainly from v3, which is the most expressed isoform in tumour and normal tissues presenting a cytosolic distribution [3]. Consideration of ROR1 v1 expression in MCL, DLBCL and FL is a crucial topic for an accurate biomarker trial patient selection strategy to assure efficacy of new therapeutic modalities targeting ROR1 such as ADC, TCE, or CAR-T, as their mode-of-actions relies on the presence of membrane tumour target [1, 10]. A detailed characterization of ROR1 v1 protein expression in normal tissues will be relevant to clarify potential ROR1-related toxicities during safety target assessment. The number of ROR1 molecules present in the cell membrane per tumour cell, as well as the minimum amount of ROR1 positive tumour cells per cancer lesion required to secure therapeutic success are relevant aspects that cannot be addressed by this kind of prevalence analysis. The H-scores calculated for each ROR1 IHC positive case were presented as waterfall graphs in Fig 2 showing the heterogeneity of each haematological indication regarding levels of ROR1 expression. This heterogeneity suggests that any potential trial patient selection strategy for MCL, DLBCL and FL should include ROR1 positive cases and exclude ROR1 negative ones to assure successful development of new therapeutic modalities targeting ROR1. The medium-to-low ROR1 IHC prevalence results we are showing in these three indications may be influenced by particularities from the tissue donors involved in our cohorts, such as treatment, ethnicity, disease stage, etc. An increase in the number of analyzed cases may not dramatically change these prevalence values because the number of cases used were comparable to the number of cases used in other IHC prevalence studies that accurately described different tumour targets [12, 13]. Also, the number of cases shown in Table 1 reflect a significant effort that allow the collection and analysis of these difficult to obtain human samples during a reasonable period of time. In addition, the development of a new v1-specific anti-ROR1 Ab for IHC should reduce the amount of ROR1 tumour staining, which may impact it prevalence in MCL, DLBCL and FL by reducing the values shown at this point. Our results from AML and T-NHL were mostly negative for ROR1 protein levels via IHC, so a more restrictive v1-specific anti-ROR1 Ab will not change the IHC prevalence data presented here for these two indications. Of note, our ROR1 IHC prevalence results are lower than the results presented by another study performed via FC where ROR1 tumour membrane median positivity for MCL was 56%; DLBCL 50%; FL 28%; and AML 36% [5]. This study was conducted with a different anti-ROR1 clone 1A8, of which the specificity/sensitivity towards peptides expressed by v1 and/or 3 is unclear, when compared to our anti-ROR1 clone 6D4. The aforementioned study’s results are presented as median values from ROR1 positive tumour cells, not averages, which could contribute to the differences. Finally, FC and IHC are methodologies that may differ in sensitivity, an additional aspect that may also contribute to this discrepancy.

The ROR1 FC data generated from CLL and HCL samples was based only on a membrane signature considering that membrane distribution is required to assess ROR1 as a tumour target for new therapeutic modalities such as ADC, TCE, or CAR-T [1, 10]. All CLL and HCL analyzed cases were positive for ROR1 with different levels of expression as presented in Fig 3. These results are well-aligned with previously FC published data where all CLL and HCL cases were also ROR1 positive [5]. However, our data set presented larger dynamic ranges of positivity in both CLL and HCL cohorts, which can be explained by the fact that both of our investigated disease groups are larger in number of cases, representing both disease populations better. More extensive CLL studies have already shown that approximately 5% of CLL patients are ROR1 negative [16], and due to its high prevalence in CLL, ROR1 is currently used via FC to refine the diagnosis of this disease [10, 17]. The number of HCL cases in the previous publication was only 2 [5], while we analyzed 14 cases, indicating that low numbers of HCL patients reflects how challenging it is to obtain samples from such a rare disease type. This highly positive ROR1 membrane profile in CLL and HCL could help to avoid patient selection for trial recruitment in case ROR1 is used as tumour target for ADC, TCE, or CAR-T development [1, 10]. Another potential advantage is that a trial biomarker strategy without patient selection could profit from a direct focus on the detection of a ROR1 cut-off that correlates with drug response. Finally, an additional benefit from using a FC multiplexing platform in our study, is that it allowed us to extend the ROR1 analysis to additional disease-related parameters, such as: B-cell lineage analysis using biomarkers like CD20; a clonality investigation via Kappa/Lambda ratio; and an immunophenotypic aberrancy characterization via CD5, CD22, CD25 and CD103 staining. Our results confirmed the expected disease profile in CLL and HCL for these 3 parameters as reviewed elsewhere [14, 15].

To compare ROR1 prevalence across these investigated leukaemias and lymphomas, we computed ROR1 positivity from each indication as positive or negative, which was the only common parameter that could be identified from both IHC and FC analysis. It is understood that the scope of this study does not encompass the sensitivity between IHC and FC methods, which could restrain their comparison. In this study, we clearly identified three groups of diseases. The first group, showing high levels of ROR1, includes CLL and HCL, with significant differences from the other six indications (Fig 4). The second group, with medium-to-low levels of ROR1, includes MCL, TNBC, DLBCL and FL. In this second group, MCL and TNBC were the indications with the highest average levels of ROR1, showing significant differences when compared to the third group. The third group, with ROR1 negative disease, includes AML and T-NHL. TNBC was used as a reference solid tumour indication for the significant amount of ROR1 present [7]. TNBC has already been considered as a lead indication in trials with new anti-ROR1 therapeutic modalities [10].

In conclusion, ROR1 protein expression data from these investigated haematological indications has a heterogeneous prevalence, suggesting that ROR1 as a tumour target for novel therapies should be considered with different biomarker strategies for patient selection pending on levels of tumoral ROR1 positivity.

## Supporting information

S1 Fig A) AF647 fluorochrome-labelled anti-human ROR1 clone selection for FC analysis. Comparison performance between different AF647 fluorochrome-labelled Ab for the detection of ROR1 via FC in ROR1+ cell line Kasumi-2. Data from directly AF647 labelled anti-ROR1 clones such as rbXBR1-402, Hu4-2-17-LC and 2A2 as well as unlabeled rbXBR1-402 plus a secondary AF647-labelled Ab is presented. B) Panel marker used for tumoural B-cell FC selection.
(DOCX)

S1 File Raw data Fig 3. (XLSX)

S2 File Raw data Fig 4. (XLSX)

## References

[1] Borcherding N, Kusner D, Liu GH, Zhang W. ROR1, an embryonic protein with an emerging role in cancer biology. Protein Cell. 2014;5: 496–502. doi: 10.1007/s13238-014-0059-7 24752542 PMC4085287

[2] John M, Ford CE. Pan-tissue and -cancer analysis of ROR1 and ROR2 transcript variants identify novel functional significance for an alternative splice variant of ROR1. Biomedicines. 2022;10: 2559. doi: 10.3390/biomedicines10102559 36289823 PMC9599429

[3] Baskar S, Kwong KY, Hofer T, Levy JM, Kennedy MG, Lee E, et al. Unique cell surface expression of receptor tyrosine kinase ROR1 in human B-cell chronic lymphocytic leukemia. Clin Cancer Res. 2008;14: 396–404. doi: 10.1158/1078-0432.CCR-07-1823 18223214

[4] Daneshmanesh AH, Mikaelsson E, Jeddi-Tehrani M, Bayat AA, Ghods R, Ostadkarampour M, et al. Ror1, a cell surface receptor tyrosine kinase is expressed in chronic lymphocytic leukemia and may serve as a putative target for therapy. Int J Cancer. 2008;123: 1190–1195. doi: 10.1002/ijc.23587 18546292

[5] Daneshmanesh AH, Porwit A, Hojjat-Farsangi M, Jeddi-Tehrani M, Tamm KP, Grandér D, et al. Orphan receptor tyrosine kinases ROR1 and ROR2 in hematological malignancies. Leuk Lymphoma. 2013;54: 843–850. doi: 10.3109/10428194.2012.731599 22988987

[6] Hudecek M, Schmitt TM, Baskar S, Lupo-Stanghellini MT, Nishida T, Yamamato TN, et al. The B cell tumour-associated antigen ROR1 can be targeted with T cells modified to express a ROR1- specific chimeric antigen receptor. Blood. 2010;116: 4532–4541. 20702778 10.1182/blood-2010-05-283309 PMC2996114

[7] Balakrishnan A, Goodpaster T, Randolph-Habecker J, Hoffstrom BG, Jalikis FG, Koch LK, et al. Analysis of ROR1 protein expression in human cancer and normal tissues. Clin Cancer Res. 2017;23: 3061–3071. doi: 10.1158/1078-0432.CCR-16-2083 27852699 PMC5440207

[8] Zhang S, Chen L, Wang-Rodriguez J, Zhang L, Cui B, Frankel W, et al. The onco-embryonic antigen ROR1 is expressed by a variety of human cancers. Am J Pathol. 2012;181: 1903–1910. doi: 10.1016/j.ajpath.2012.08.024 23041612 PMC3509760

[9] Ghaderi A, Daneshmanesh AH, Moshfegh A, Kokhaei P, Vågberg J, Schultz J, et al. ROR1 is expressed in diffuse large B-cell lymphoma (DLBCL) and a small molecule inhibitor of ROR1 (KAN0441571C) induced apoptosis of lymphoma cells. Biomedicines. 2020;8,170. doi: 10.3390/biomedicines8060170 32586008 PMC7344684

[10] Kipps TJ. ROR1: an orphan becomes apparent. Blood. 2022;140: 1583–1591. doi: 10.1182/blood.2021014760 35580162 PMC10653015

[11] Kreitman RJ, Arons E. Up-date on hairy cell leukemia. Clin Adv Hematol Oncol. 2018;16: 205–215. 29742076 PMC6290912

[12] Silva MA, Ryall K, Wilm C, Caldara J, Grote HJ, Patterson-Kane JC. PD-L1 immunostaining scoring for non-small cell lung cancer based onimmunosurveillance parameters. PLOS ONE. 2018;13: e0196464. doi: 10.1371/journal.pone.0196464 29874226 PMC5991369

[13] Silva MA, Triltsch N, Leis S, Kanchev I, Tan TH, Van Peel B, et al. Biomarker recommendation for PD-1/PD-L1 immunotherapy development in pediatric cancer based on digital image analysis of PD-L1 and immune cells. J Pathol Clin Res. 2020;6: 124–137. doi: 10.1002/cjp2.152 31922656 PMC7164376

[14] Kroft SH, Harrington AM. Flow cytometry of B-cell neoplasms. Clin Lab Med. 2017;37: 697–723. doi: 10.1016/j.cll.2017.07.001 29128065

[15] Cherian S, Hedley BD, Keeney M. Common flow cytometry pitfalls in diagnostic hematopathology. Cytometry. 2019;96B: 449–463. doi: 10.1002/cyto.b.21854 31697047

[16] Cui B, Ghia EM, Chen L, Rassenti LZ, DeBoever C, Widhopf GF 2nd, et al. High-level ROR1 associates with accelerated disease progression in chronic lymphocytic leukemia. Blood. 2016;128: 2931–2940. doi: 10.1182/blood-2016-04-712562 27815263 PMC5179332

[17] Arandas de Sousa F, Magalhães Millan N, Patussi Correia R, da Costa Vaz A, Schimidell A, Carmona Miyamoto P, et al. ROR1 expression in mature B lymphoid neoplasms by flow cytometry. Cytometry B Clin Cytom. 2024;106: 74–81. doi: 10.1002/cyto.b.22157 38273649

