# Supplementary material for: Tumour assessment of ROR1 levels in various adult leukaemia and lymphoma types
Source: PLoS One. 2024 Nov 4;19(11):e0313026. doi: 10.1371/journal.pone.0313026 (PMC11534244; doi:10.1371/journal.pone.0313026)
Supplement: S1 Fig — A) AF647 fluorochrome-labelled anti-human ROR1 clone selection for FC analysis. Comparison performance between different AF647 fluorochrome-labelled Ab for the detection of ROR1 via FC in ROR1+ cell line Kasumi-2. Data from directly AF647 labelled anti-ROR1 clones such as rbXBR1-402, Hu4-2-17-LC and 2A2 as well as unlabeled rbXBR1-402 plus a secondary AF647-labelled Ab is presented. B) Panel marker used for tumoural B-cell FC selection. (DOCX) [file pone.0313026.s001.docx]

**Tumor marker panel for CLL and HCL**

**A**

| **Labelled Ab** | CD5 ECD | CD19 pacific blue | CD20 PC5.5 | CD22 APC-A700 | CD25  APC-A750 | CD45 BV510 | CD103 FITC | Kappa PE | Lambda PC7 | ROR1 AF647 |
| --- | --- | --- | --- | --- | --- | --- | --- | --- | --- | --- |
| **Clone (Vendor)** | BLA1a  (BC) | J3-119  (BC) | B9E9  (BC) | SJ10.1H11  (BC) | B1.49.9  (BC) | HI30  (BD) | 2G5  (BC) | Polyclonal  (Dako) | Polyclonal  (BC) | Hu4-2-17-LC  (BI) |
| **Concentrations** | 0,2 | 0,4 | 0,1 | 0,2 | 1,7 | 6,7 | 6,7 | 4,4 | 0,2 | 0,2 |

BC: Beckman Coulter; BD: BD Biosciences; BI: Boehringer-Ingelheim

**B**
